# Basal-like breast cancer: molecular profiles, clinical features and survival outcomes

**DOI:** 10.1186/s12920-017-0250-9

**Published:** 2017-03-28

**Authors:** Heloisa H. Milioli, Inna Tishchenko, Carlos Riveros, Regina Berretta, Pablo Moscato

**Affiliations:** 1 Centre for Bioinformatics, Biomarker Discovery and Information-Based Medicine, Hunter Medical Research Institute, Lot 1, Kookaburra Circuit, New Lambton Heights, 2305 Australia; 2 School of Environmental and Life Sciences, The University of Newcastle, University Drive, Callaghan, 2308 Australia; 3 School of Electrical Engineering and Computer Science, The University of Newcastle, University Drive, Callaghan, 2308 Australia; 4 CReDITSS Unit, Hunter Medical Research Institute, Lot 1, Kookaburra Circuit, New Lambton Heights, 2305 Australia

**Keywords:** Breast cancer, Intrinsic subtypes, Basal-like, Triple-negative, Molecular profile, Survival outcome, Gene expression, Signature, Copy number aberration, MicroRNA

## Abstract

**Background:**

Basal-like constitutes an important molecular subtype of breast cancer characterised by an aggressive behaviour and a limited therapy response. The outcome of patients within this subtype is, however, divergent. Some individuals show an increased risk of dying in the first five years, and others a long-term survival of over ten years after the diagnosis. In this study, we aim at identifying markers associated with basal-like patients’ survival and characterising subgroups with distinct disease outcome.

**Methods:**

We explored the genomic and transcriptomic profiles of 351 basal-like samples from the METABRIC and ROCK data sets. Two selection methods, labelled *Differential* and *Survival* filters, were employed to determine genes/probes that are differentially expressed in tumour and control samples, and are associated with overall survival. These probes were further used to define molecular subgroups, which vary at the microRNA level and in DNA copy number.

**Results:**

We identified the expression signature of 80 probes that distinguishes between two basal-like subgroups with distinct clinical features and survival outcomes. Genes included in this list have been mainly linked to cancer immune response, epithelial-mesenchymal transition and cell cycle. In particular, high levels of *CXCR6*, *HCST*, *C3AR1* and *FPR3* were found in Basal I; whereas *HJURP*, *RRP12* and *DNMT3B* appeared over-expressed in Basal II. These genes exhibited the highest betweenness centrality and node degree values and play a key role in the basal-like breast cancer differentiation. Further molecular analysis revealed 17 miRNAs correlated to the subgroups, including hsa-miR-342-5p, -150, -155, -200c and -17. Additionally, increased percentages of gains/amplifications were detected on chromosomes 1q, 3q, 8q, 10p and 17q, and losses/deletions on 4q, 5q, 8p and X, associated with reduced survival.

**Conclusions:**

The proposed signature supports the existence of at least two subgroups of basal-like breast cancers with distinct disease outcome. The identification of patients at a low risk may impact the clinical decisions-making by reducing the prescription of high-dose chemotherapy and, consequently, avoiding adverse effects. The recognition of other aggressive features within this subtype may be also critical for improving individual care and for delineating more effective therapies for patients at high risk.

**Electronic supplementary material:**

The online version of this article (doi:10.1186/s12920-017-0250-9) contains supplementary material, which is available to authorized users.

## Background

Approximately 15% of all breast cancer cases are of basal-like subtype, often aggressive and highly recurrent lesions [1–3]. Basal-like breast cancers (BLBCs) are defined by the lack of expression of the hormone receptors oestrogen (ER) and progesterone (PR), and the human epidermal growth factor receptor-2 (*HER2*) [4, 5]. Histologically, these tumours show high grade, high mitotic indices, presence of central necrotic or fibrotic zones, pushing borders of invasion, lymphocytic infiltrate and atypical medullary features [6]. The breast basal cell layer is also characterised by high expression of cytokeratins (CK5/6, CK14, and CK17) and epidermal growth factor receptor (*EGFR*), amongst other markers [7–11]. All these features contribute to the limited therapeutic response and therefore impact in the refractory nature of these tumours [12, 13]. Thus, patients diagnosed with BLBC have a poor prognosis and a short-term disease-free and overall survival [14]. A better understanding of the pathophysiology and molecular basis of basal-like tumours is necessary to delineate patient outcomes.

At the molecular level, basal-like tumours are considered more homogeneous than the immunohistochemically defined triple-negative breast cancers (TNBCs), even though the terminologies are used interchangeably [1, 15]. Despite the relative molecular homogeneity, patients within this group still show divergent disease outcomes [12, 14, 16]: some patients show high mortality and recurrence rates within the first 3-5 years, in contrast to others who survive over 10 years – with no recurrence – following the diagnosis [12, 14, 16]. For the latter group, the prognosis is better than those of luminal breast cancer subtype [8, 17]. These observations suggest that BLBCs may be composed of at least two clinically distinct groups, with poor or excellent survival [10]. The molecular characterisation of these basal-like tumours is of particular interest in medicine since it may bring new insights to the disease understanding and management. Identifying markers and mechanisms involved in the differentiation of BLBCs is therefore an essential progression towards this end. Moreover, it would allow the development of tailored treatments with more effective individual response, leading to more personalised and conservative interventions for breast cancers [18].

Recent investigation of TNBCs pointed to the existence of intrinsic basal-like subtypes, with distinct molecular patterns [19–21]. The stratification performed and described by Lehmann et al. (2011) [19] revealed the involvement of enriched cell cycle and cell division components in Basal-like 1 (BL1); growth factor signalling, glycolisis and gluconeogenesis pathways in Basal-like 2 (BL2); and immune cell processes in Immunomodulatory (IM). The authors also determined two other groups partially overlapping the basal-like subtype defined by the PAM50 classifier [22]: Mesenchymal (M) and Mesenchymal stem-like (MSL). Alternatively, Burstein and colleagues [20] defined the Basal-Like Immune-Suppressed (BLIS) and Basal-Like Immune-Activated (BLIA) subtypes. The former tumour type is characterised by multiple SOX family transcription factors, while the latter is described by Stat signal transduction molecules and cytokines. More recently, Jézéquel et al. (2015) [21] pointed to two other groups: a basal-like with low immune response and high M2-like macrophages, and a basal-enriched with high immune response and low M2-like macrophages. All studies above described have focused on investigating the molecular heterogeneity of TNBCs, partially supporting each other.

Multi-gene models have also been applied to predict breast cancer subtype [22, 23], recurrence [24] and survival [25, 26]. The selection of genes across samples has generally been associated with hormonal expression levels and proliferation modules. Since BLBCs and TNBCs are hormone receptor (ER and PR) negative and highly proliferative, the prediction power of markers to further separate patients at risk within these groups is of limited value in the current models [27]. Clinical assays independently modelling triple-negative samples have revealed superior ability in predicting outcomes of early stage tumours [28, 29]. These assays and most approaches, however, have focused on the immunohistochemically defined TNBCs [10, 30, 31]. A more robust approach for characterising BLBC outcomes is yet to be developed. Accordingly, a proper investigation of BLBCs remains mandatory and determinant for patients diagnosed within this subtype [9].

As the classification of TNBCs is not an ideal surrogate for defining BLBCs entities, a characterisation of basal-like tumours at the genomic and transcriptomic levels is an urgent need. In this contribution, we aim at identifying markers associated with patients’ survival using larger breast cancer cohorts from the Molecular Taxonomy of Breast Cancer International Consortium (METABRIC) [32] and Research Online Cancer Knowledgebase (ROCK) [33]. Through the determination of this signature, our objective is to stratify 351 tumours into basal-like subgroups, with varying clinical features and survival outcomes, and further describe each of them. Accordingly, we plan to explore the microarray data – including gene (mRNA) and microRNA (miRNAs) expression values, and copy number aberration (CNA) measurements – to expand the molecular characterisation of BLBCs, which to our knowledge has not yet been performed. The assessment of more comprehensive profiles of BLBCs is relevant for defining groups-at-risk in clinical settings and, more importantly, for improving therapy response.

## Methods

### Breast cancer data sets

The METABRIC genomic and transcriptomic data sets were downloaded from the European Genome-Phenome Archive (EGA) (http://www.ebi.ac.uk/ega), under the accession numbers EGAS00000000083 and EGAS00000000122. These publicly available collections contain genotyping (Affymetrix SNP 6.0), log_2_ normalised gene expression (Illumina_Human_WG-v3) and miRNA expression (Agilent ncRNA 60k) arrays for over 2000 breast tumours and 144 control (non-tumour) breast samples [32]. The original METABRIC study was approved by the ethics Institutional Review Boards in the UK and Canada (Addenbrooke’s Hospital, Cambridge, United Kingdom; Guy’s Hospital, London; Nottingham; Vancouver; Manitoba). Further analysis on this data was approved by the Human Research Ethics Committee (HREC) at the University of Newcastle, Australia (approval number: H-2013-0277).

The METABRIC cohort has a comprehensive description of patients long-term clinical and pathological outcomes. Tumour samples were assigned to a breast cancer subtype (luminal A, luminal B, HER2-enriched, normal-like, or basal-like) using an ensemble learning approach [34], employing the set of 50 genes defined by Parker et al. (2009) [22]. This approach has been previously shown to improve the samples classification and subtypes’ assignement in METABRIC data set, and has revealed more consistency in terms of clinical features and survival outcomes [34]. Based on these labels, a subset of 250 basal-like tumours was selected for analysis in this study. For training and test purposes, this subset was randomly split into two sets of equal size (125) to avoid possible bias from the original cohort. The sets are hereafter referred to as the *training* and *validation* sets.

For additional validation across platforms, we used the ROCK data set obtained at Gene Expression Omnibus (GEO) (http://www.ncbi.nlm.nih.gov/geo/), under data source number GSE47561 [33, 35]. This data set integrates ten different studies (GSE2034, GSE11121, GSE20194, GSE1456, GSE2603, GSE6532, GSE20437, GSE7390, GSE5847 and E-TABM-185) performed on the Affymetrix HG-U133A technology. The compiled matrix contains log_2_ RMA renormalised gene expression values for 1570 tumour samples, 101 of which are of basal-like subtype. The ROCK data set includes representative information for survival analysis, however, it lacks standard clinicopathological data which therefore has not been considered in this study.

### Probe selection approach

Since the first aim of our study is to identify markers driving survival among basal-like patients, we designed a filtering technique to select a representative probe signature and reduce the bias arising from the high number of probes (48,803) and low number of samples (125) in the training set. We defined two relevant criteria to select probes, which are involved in tumour initiation and/or progression, and are also correlated to survival, as detailed below.

The *Differential* filter [36] was employed to select probes exhibiting distinct expression levels between tumours and controls. The underlying assumption is that probes truly correlated with breast cancer are linked to genomic changes or variations from healthy to cancerous tissue. We applied the *Differential* filter to each of the 48803 probes to test their separation power between the 125 tumours and 144 controls. This filter tests for three feasible cases: the expression levels in tumours are (a) *lower than*, (b) *higher than*, or (c) *lower and higher than* in control samples. The last case refers to genes that are up-regulated in some tumours and down-regulated in others, while the expression levels of controls lie between these two groups. To calculate a *p*-value for this case, we mirrored all expression levels on one side with respect to the mean value of controls. The separation power of each probe was defined as the minimal Wilcoxon test *p*-value calculated for the three cases. To determine the number of probes passing the *Differential* filter, we plotted the ordered log10-normalised *p*-values against the corresponding probe ranks. The threshold was set approximately at the point of the highest curvature of this function. This threshold is based on the naturally emerging systemic behaviour and does not require an external definition. Probes passing this filter are referred to as the *differential probe set*.

The *Survival* filter [36] was used to further identify probes for which the expression levels are associated with patients’ survival. This filter employs the Kaplan-Meier estimator to compute the survival probabilities. The stratification power of each probe is calculated using the Log-rank test applied to two groups of samples corresponding to quantiles with the lowest and the highest expression values, respectively. We defined these quantiles by ordering all samples by their expression values of a probe and selected samples in the first and last thirds (the quantile from 0 to 33% in the relatively under-expressed and from 67 to 100% in the relatively over-expressed group). This analysis was performed in *R* using the package *survival* [37]. Since the survival information is not provided for all samples, this calculation was based on 115 basal-like tumour samples (from the total of 125) in the METABRIC training set. To determine the number of probes passing the *Survival* filter we used a similar threshold definition as for the *Differential* approach, i.e. by ordering the log10-normalised *p*-values that emerged from the Log-rank test. These probes are further referred to as the *survival probe set*.

### Clustering basal-like tumour samples

The second aim of our study is to identify and characterise basal-like subgroups with varying disease outcomes. To this end, we performed a hierarchical clustering of samples based on the previously defined *survival probe set*. This procedure exploits the assumption that probes showing most variations in expression and co-expression among each other are involved in similar biological mechanisms and have a high impact on the groups delineation. To calculate the dissimilarity between the 115 samples from the METABRIC training set, for which the survival information is provided, we used the square root of the Jensen-Shannon divergence [38–40]. We then generated the hierarchical clustering with the Ward’s criterion that minimises the variance within clusters, using the *R* package *stats* [41].

We further examined which probes from the *survival probe set* contribute the most to the separation of basal-like subgroups using the Wilcoxon test. We then ordered the log10-normalised *p*-values to determine the probes that significantly differentiate between the subgroups by using the same threshold criterion as for the *Differential* filter. The purpose of this procedure is to refine the probes that best segregate basal-like subgroups of distinct disease outcome. These probes are further referred to as the *probe signature* and expose striking genes and cell mechanisms involved in the subgroups differentiation.

### Validation across data sets

The basal-like entities were first matched to the METABRIC validation set by means of centroids computed based on the previously defined *probe signature*. Samples in this data set were then assigned to a subgroup according to the minimal Euclidean distance to a centroid.

An external validation was conducted on the ROCK data set, for which the centroids were mapped across technologies – from Illumina to Affymetrix – using the gene annotation packages *hgu133a.db* and *illuminaHumanv3.db* [42] in *R* *Bioconductor*. Since the mRNA level measurement and normalisation differ between METABRIC (Illumina) and ROCK (Affymetrix) data sets, we standardised the calculated centroid absolute values with respect to the average expression levels computed for all basal-like samples. This procedure is depicted in Eq. 1, where *s*_i,j_ is the expression value of probe *j* for sample *i*, and *N* is the total number of basal-like samples (*N* is equal to 115 in the METABRIC training set).

1 $$ s_{i,j}^{\text{standard}} = \frac{s_{i,j}}{ \frac{1}{N} \sum_{i=1}^{N} s_{i,j}}  $$

Following the centroids’ normalisation, an analogous transformation of Affymetrix gene expression values was necessary to enable their direct application. Thus, we applied the same formula (Eq. 1) to the ROCK data set, where the number *N* of total samples is 101. The assignment to subgroups was based on the minimal Euclidean distance to a standardised centroid.

### Network analysis

With the purpose to identify key players within the *probe signature* and their relation to each other, we generated and plotted a network graph using the Minimum Spanning Tree (MST) [43]. The distance *d*(*x*,*y*) between two probes *x* and *y* were defined as *d*(*x*,*y*)=1−|*ρ*_*S*_(*x*,*y*)|, where *ρ*_*S*_(*x*,*y*) is the value of the Spearman correlation between the probe expression calculated for 125 tumour samples from the training set. To quantify the network analysis, we computed the betweenness centrality and node degree of each node (probe) using the package *igraph* [44] in *R*.

Generally, nodes with high betweenness centrality and degree values represent potential key players within the network. With regards to the centrality values, the most representative entities are highly connected to the rest of the tree; leaf-nodes have a betweenness centrality value of 0, while the most traversed nodes are assigned with the highest values (normalised up to 1). Node degree, on the other hand, is indicative of the number of direct neighbours of a node. Thus, probes with high degrees are also central (representative) for local groups with a relatively strong probe co-expression.

### MicroRNA differential expression

To uncover the miRNAs differentiating the most between the basal-like subgroups, we applied the Wilcoxon test to expression values of each of the 853 probes available in the METABRIC data set. We considered those miRNAs with the emerging *p*-values smaller than 0.01 in both training and validation sets, as relevant for the separation between the subgroups. Both data sets were used due to the limited number of samples (146 in total) for which the miRNA expression profiles were provided. The miRNA probes were further investigated for possible target genes within the *probe signature* using *R* *Bioconductor* (*RmiR.Hs.miRNA* [45]) across five databases: *miRBase*, *TarBase*, *PicTar*, *MirTarget2* and *miRanda*. For the miRNA and gene annotation we used the packages *hgug4112a.db* [46] and *illuminaHumanv3.db* [42], respectively.

### Copy number aberration profiles

To quantise the CNA information we employed the cytobands defined in the hg18 data base that corresponds to the METABRIC platform. Aberrations were divided into two categories: losses (originally denoted as homozygous and heterozygous deletions) and gains (gains and amplifications). For each basal-like subgroup we then calculated the occurrence rates of gains and losses per cytoband, and applied the Binomial test to examine the hypothesis that the CNA distributions were the same among patient subgroups.

We further calculated the Percent Genome Altered (PGA) for each of the basal-like subgroups and applied the Wilcoxon test to these rates to obtain a significance value of the difference between them. The aim of this approach is to identify stable/unstable genome profiles associated with the patient subgroups defined by our *probe signature* and to statistically describe whether they are consistently diverging.

## Results

### Survival-related probes defining basal-like breast cancer subgroups

With the application of the *Differential* and *Survival* filters in the METABRIC training set – as detailed in “Methods” – we identified 15000 and 400 probes related to cancer initiation and/or progression, and patients survival, respectively. The corresponding probes in the *differential probe set* with distinct expression levels between tumours and controls showed significant *p*-values ranging from 2.36·10^−45^ to 1.53·10^−7^. The reduced number of probes in the *survival probe set* related to the individual survival had significant *p*-values ranging from 1.11·10^−4^ to 0.038. These probes, ultimately, comprise a representative signature driving the outcome of basal-like patients in the METABRIC breast cancer cohort.

**Fig. 1:**
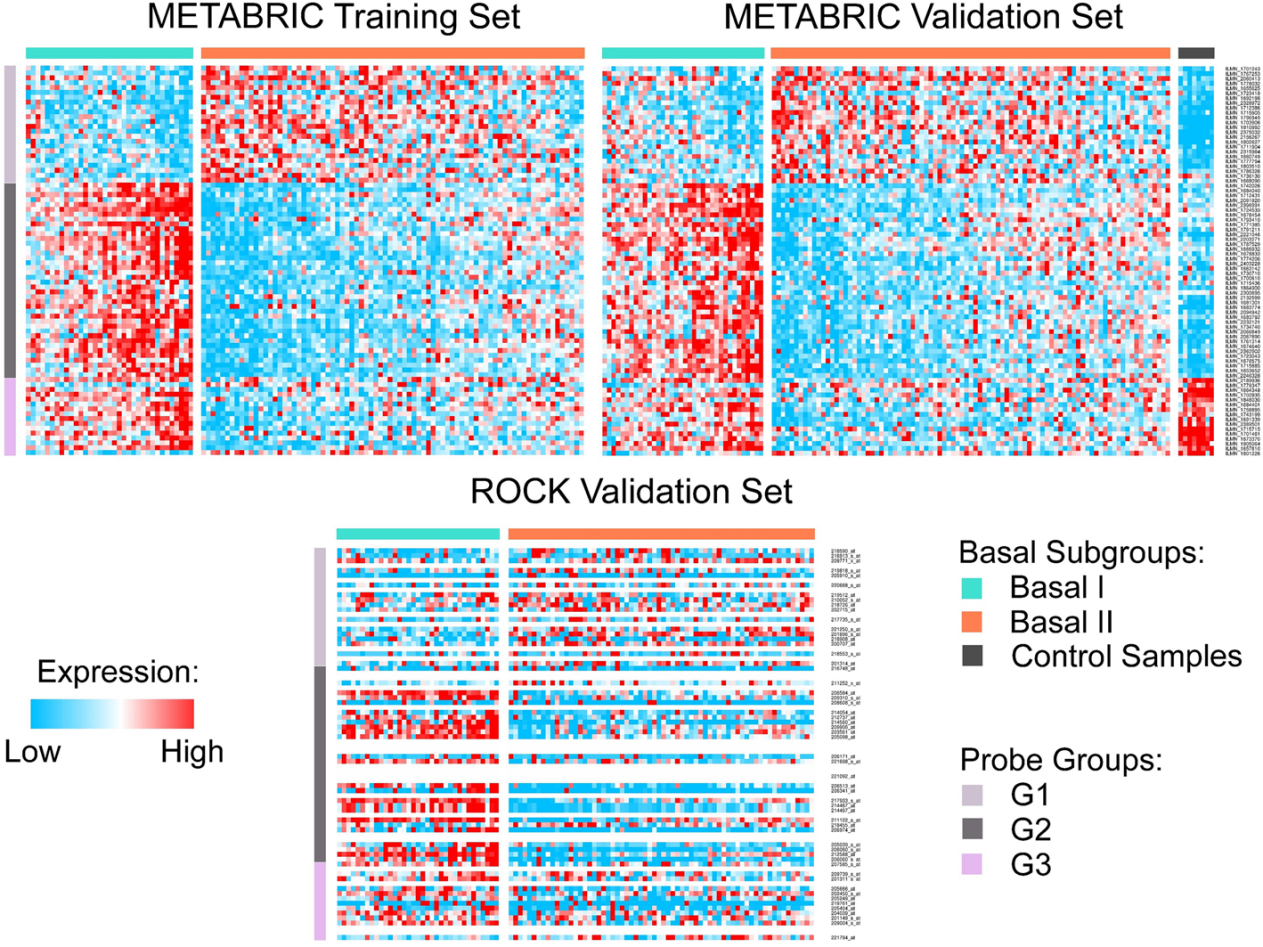
Heat map of the 80-probe signature in METABRIC training set. This figure displays 80 survival-related probes clustered by their mutual correlation. Samples in each basal-like subgroup are ordered by their overall rank and the expression values are normalised across individuals. The subgroups in the METABRIC validation set were defined using centroids computed in the training set. In the ROCK data set, 55 Affymetrix probes matched the 80 Illumina signature; samples in this data set are ordered by their overall rank within each subgroup

The hierarchical clustering of 115 basal-like samples based on the *survival probe set* has revealed two major subgroups: Basal I and Basal II (Additional file 1: Figure S1). A separation into more than two subgroups – in the next and subsequent hierarchical divisions in the dendrogram – was not supported due to the high similarity of subgroups in terms of their molecular profile and clinical outcome. The application of the Wilcoxon test has defined the *probe signature* containing the top 80 probes, with significant *p*-values ranging from 1.75·10^−13^ to 3.77·10^−4^, differentiating the most between the two basal-like groups at the transcriptomic (mRNA) level. A heat map of the 80-probe signature for the training set is plotted in Fig. 1, where samples are ordered within each subgroup by their Euclidean distance to the corresponding centroids (Additional file 2: Tables S1, S2 and S3).

To characterise the 80-probe signature with respect to their cellular function, we clustered the probes by their mutual correlation into three groups (Table 1) – G1, G2 and G3 – and annotated using the Database for Annotation, Visualization and Integrated Discovery (DAVID) (Additional file 3: Tables S4, S5 and S6). This analysis revealed that G1 probes are strongly associated with cell cycle control and cell division; they are over-expressed in Basal II subgroup. G2 showed relation to immune system and inflammatory response. Remarkably, the expression levels of G2 probes in Basal II are similar to that observed in controls, but much higher in Basal I, suggesting an intratumoral infiltration by lymphocytes in this subgroup. In the last group, G3, probes indicate an association (not significant) with metal-binding processes; they are under-expressed in Basal II when compared to Basal I and control samples.

**Table 1:** The 80-probe signature related to survival

Gs	Gene	Probe ID	B	ND
G1	*C10orf2*	ILMN_1701243	0.17	3
	*RRP12*	ILMN_1767253	0.12	4
	*CD24*	ILMN_2060413	0	1
	*SURF6*	ILMN_1778032	0	1
	*GPATCH1*	ILMN_1655625	0.03	2
	*CEL*	ILMN_1723418	0	1
	*LOC641765*	ILMN_1692198	0	1
	*DNMT3B*	ILMN_2328972	0.1	4
	*MIS18A*	ILMN_1712386	0	1
	*DSN1*	ILMN_1715905	0.03	2
	*TPX2*	ILMN_1796949	0.14	3
	*HJURP*	ILMN_1703906	0.42	5
	*CAD*	ILMN_1810992	0	1
	*BEND3*	ILMN_2375032	0.21	3
	*EIF2AK1*	ILMN_2156267	0.07	2
	*PSMG3*	ILMN_1802627	0.47	3
	*MXD3*	ILMN_1711904	0	1
	*PSRC1*	ILMN_2315964	0	1
	*ASPSCR1*	ILMN_1660749	0.05	2
	*PRKCSH*	ILMN_1777794	0.03	2
	*LOC650803*	ILMN_1803510	0.05	2
	*KCTD15*	ILMN_1786326	0	1
	*RBFA*	ILMN_1736130	0	1
	*STK25*	ILMN_1668090	0.03	2
G2	*PYHIN1*	ILMN_1742026	0.05	3
	*THEMIS*	ILMN_1684040	0	1
	*PCED1B*	ILMN_1712431	0.03	2
	*PTCRA*	ILMN_2091920	0	1
	*HCST*	ILMN_2396991	0.57	6
	*LY96*	ILMN_1724533	0.45	3
	*CASP4*	ILMN_1678454	0	1
	*SNTB1*	ILMN_1793410	0	1
	*GBP4*	ILMN_1771385	0.46	2
	*DOK2*	ILMN_1791211	0	1
	*GM2A*	ILMN_2221046	0	1
	*FPR3*	ILMN_2203271	0.17	4
	*C3AR1*	ILMN_1787529	0.47	7
	*FCGR2A*	ILMN_1666932	0.12	2
	*CCR1*	ILMN_1678833	0	1
	*LOC647108*	ILMN_1774206	0.03	2
	*CLEC12A*	ILMN_2403228	0	1
	*CLEC12A*	ILMN_1663142	0.03	2
	*ADORA3*	ILMN_1730710	0	1
	*CLEC7A*	ILMN_1700610	0.03	2
	*LOC650799*	ILMN_1715436	0	1
	*MIAT*	ILMN_1864900	0	1
	*IKZF3*	ILMN_2300695	0	1
	*ANKRD22*	ILMN_2132599	0.45	2
	*AIM2*	ILMN_1681301	0.03	2

**Table 1:** The 80-probe signature related to survival (*Continuation*)

Gs	Gene	Probe ID	B	ND
	*IL2RA*	ILMN_1683774	0	1
	*MARCH1*	ILMN_2094942	0.05	3
	*LAP3*	ILMN_1683792	0	1
	*GPR65*	ILMN_2232121	0.03	2
	*GPR65*	ILMN_1734740	0.05	2
	*FAM26F*	ILMN_2066849	0	1
	*CXCL11*	ILMN_2067890	0	1
	*NFS1*	ILMN_1761314	0.05	2
	*CXCR6*	ILMN_1674640	0.68	10
	*RASSF5*	ILMN_2362902	0.07	2
	*NAPSB*	ILMN_1723043	0.05	3
	*IKZF1*	ILMN_1676575	0	1
	*PTPN22*	ILMN_1715885	0	1
	*PTPRC*	ILMN_1653652	0.07	3
	*PTPN22*	ILMN_2246328	0	1
G3	*RPL36AL*	ILMN_2189936	0	1
	*GARNL3*	ILMN_1779347	0	1
	*PNPLA4*	ILMN_1664348	0	1
	*SH3BGRL*	ILMN_1702835	0.03	2
	*HS.576380*	ILMN_1848030	0	1
	*FMO1*	ILMN_1684401	0	1
	*CTSK*	ILMN_1758895	0.1	4
	*EGR2*	ILMN_1743199	0	1
	*CLEC1A*	ILMN_1691339	0	1
	*HSD11B1*	ILMN_2389501	0.03	2
	*CEBPA*	ILMN_1715715	0	1
	*TIMP3*	ILMN_1701461	0.03	2
	*FBXL5*	ILMN_1673370	0	1
	*SCARNA9*	ILMN_1805064	0	1
	*PPM1M*	ILMN_1657810	0.05	3
	*DOCK6*	ILMN_1801226	0	1

The 80 annotated Illumina probes distinguishing between basal-like subgroups are listed in this table. The official gene symbol (Gene), from UCSC Genome Browser, and Illumina probe IDs (Probe ID) are provided for each probe group (Gs), in the same order as shown in Fig. 1. This table also contains the betweenness centrality (B) and node degree (ND) values calculated for each probe in the basal-like training set

**Fig. 2:**
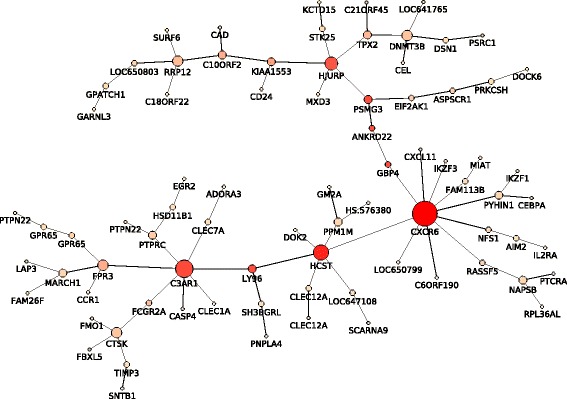
Minimum Spanning Tree of the 80-probe signature. The MST graph was generated for the 80 probes in the training set. Only probes with high correlation values between their expression levels are connected to a network. The size of each node is proportional to the computed node degree value (number of connections). The colour of each node is reflective of the betweenness centrality value ranging between low (*light pink*) and high (*red*)

The betweenness centrality and node degree analysis of the 80-probe signature (Fig. 2) further outlined important genes differentiating between Basal I and Basal II subgroups (Table 1). The genes with the highest centrality values (B ≥0.1) in G1 are *PSMG3*, *HJURP*, *BEND3*, *C10orf2*, *TPX2*, *RRP12* and *DNMT3B*; in G2, *CXCR6*, *HCST*, *C3AR1*, *GBP4*, *LY96*, *ANKRD22*, *FPR3* and *FCGR2A*; and in G3, *CTSK*. Within this set, the genes *HJURP*, *RRP12*, *DNMT3B*, *CXCR6*, *HCST*, *C3AR1*, *FPR3* and *CTSK* also showed high node degree values (ND ≥4), representative for probe co-expression, corroborating with their key role on the differentiation of basal-like carcinomas.

### Basal I and Basal II validated across independent data sets and microarray platforms

The quality of the 80-probe signature was evaluated using centroids calculated for the training set and applied to the METABRIC and ROCK validation sets. In ROCK, 55 annotated probes matched from Illumina to Affymetrix and were validated across the microarray platforms. The corresponding heat maps, in Fig. 1, showed the existence of two main basal-like subgroups, Basal I and Basal II, in both METABRIC and ROCK validation sets. The two subgroups are consistent with regards to the population size and mRNA expression levels (in G1, G2 and G3) and further support the quality of the 80-probe signature. The definition of more than two subgroups in the hierarchical clustering would lead to the separation of entities with highly similar molecular profiles.

### Clinical features and survival outcomes supporting the basal-like subgroups

The analysis of clinicopathalogical markers revealed a significant correlation between the basal-like subgroups defined in this study and tumour histology (Invasive Ductal Carcinoma versus medullary type), tumour size and p53 status (Table 2). According to histological classification, the medullary type is more common among Basal I patients. On the other hand, the Basal II subgroup is characterised by larger tumours (in size) and a higher frequency of p53 mutation. Clinical features, such as age, menopausal status (MS), grade, Nottingham Prognostic Index (NPI) and lymph nodes, did not show statistically significant variations across the two basal-like subgroups.

**Table 2:** Clinicopathological information for patients in the METABRIC data set

	Training set		Validation set	
	Basal I	Basal II	Basal I	Basal II
Age [years]				
≤ 40	7	18	4	17
41 to 50	11	18	10	21
51 to 60	8	20	9	16
> 60	9	24	13	35
mean	50.6	52.5	54.7	54.1
*p*-value	0.46		0.8	
MS				
Pre/post	18/17	36/43	15/21	37/52
Pre/post (%)	51.4%	45.6%	41.7%	41.6%
*p*-value	0.31		1	
Size [cm]				
≤ 2 cm	15	30	17	32
> 2 cm	20	50	19	55
Mean	23.5	30.6	22.1	29.6
*p*-value	0.01		0.005	
Grade				
Grade 2	2	8	5	3
Grade 3	33	71	30	85
Na	0	1	1	1
Mean	2.9	2.9	2.9	3
*p*-value	0.4		0.092	
NPI				
≤2.4	0	1	1	1
2.4 to 3.4	1	6	3	2
3.4 to 5.4	28	62	27	77
>5.4	6	11	5	9
Mean	4.7	4.6	4.5	4.6
*p*-value	0.43		0.7	
Lymph Node				
Neg/pos	16/19	37/43	17/19	47/42
Neg/pos (%)	45.7%	46.2%	47.2%	52.8%
*p*-value	1		0.34	
Histology				
ILC	0	2	0	1
IDC	28	70	23	83
IDC-med	7	5	9	3
Others	0	3	4	2
*p*-value	0.001		5.4·10^−8^	
p53				
Mut/wild	1/15	11/14	2/11	12/17
Mut/wild (%)	6.25%	44%	15.4%	41.4%
*p*-value	1.1·10^−7^		7·10^−4^	
Population size				
	35	80	36	89

The clinicopathological features described are: Age in years, menopausal status (MS), tumour Size in cm, tumour Grade [1–3], Nottingham Prognostic Index (NPI), Lymph Node invasion, histopathological classification (Histology) and p53 status, for Basal I and II subgroups in the METABRIC discovery and validation sets. In all cases, the *p*-value indicates the significance of the difference between Basal I and II subgroups. For numerical variables (Age, Size, Grade, and NPI) it was calculated using the ANOVA on ranks; for the categorical (MS, Lymph Node, Histology, p53), a binomial test was used. Population sizes for each group are indicated in the last row. Tumour histology is as follows: IDC=Invasive Ductal Carcinoma, ILC=Invasive Lobular Carcinoma, IDC-med=Medullary Carcinoma, and others include tubular, mucinous and phyllodes tumours

**Fig. 3:**
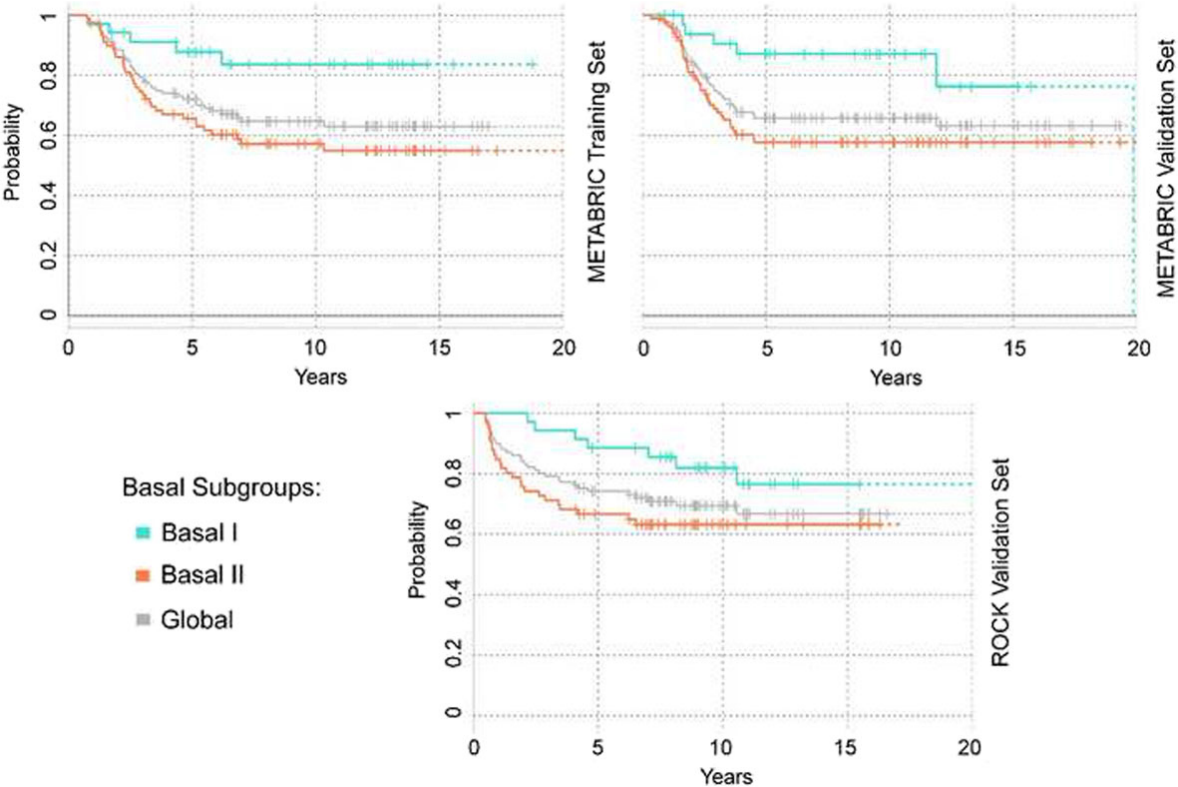
Survival curves in METABRIC and ROCK data sets. The survival analysis was performed using the Kaplan-Meier estimator. The *grey line* shows the disease specific survival of all basal-like samples in the training and validation sets, respectively. Basal I subgroup is shown in turquoise, and Basal II in coral. Ticks represent sensors of patients who are alive and drops denote deaths. Survival curves based on the last 10 observations are plotted in *dash*

The survival analysis revealed significant differences in patients’ outcome between Basal I and Basal II. Basal I showed a better prognosis in comparison to Basal II in all data sets (Fig. 3), with the Log-rank test *p*-values of 0.0097, 0.017 and 0.043 for the METABRIC training, validation and ROCK data sets, respectively.

### MicroRNAs differentially expressed between Basal I and Basal II subgroups

**Fig. 4:**
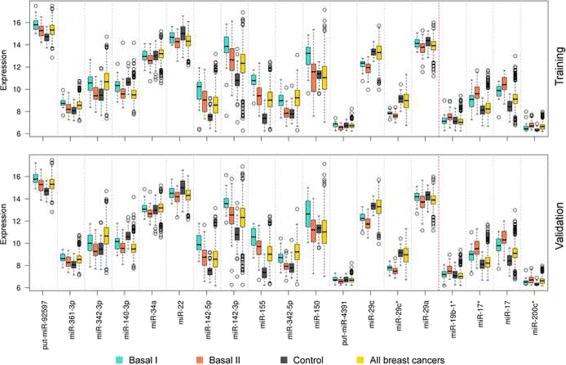
The *box* plot of miRNAs differentiating between Basal I and Basal II subgroups. The image shows the expression levels of 19 miRNAs across basal-like subgroups and other samples in the METABRIC data set. Basal I is shown in turquoise, Basal II in coral, controls in *grey* and all breast cancers in *yellow*

We identified 17 miRNAs and 2 putative probes differentially expressed between the two basal-like subgroups (Table 3), with the Wilcoxon test *p*-values smaller than 0.01 in both METABRIC data sets (Additional file 4: Tables S7, S8 and S9). The probes hsa-miR-155, -342-5p and -150 showed the lowest *p*-values and an over-expression in Basal I, when compared to Basal II and control samples. The transcripts hsa-miR-19b-1*, -17* and -200c*, on the other hand, were over-expressed in Basal II tumours relative to Basal I and controls. The expression levels of all probes are depicted in Fig. 4. Additionally, the identified miRNAs were matched against the 80-probe signature revealing a set of 50 gene-targets across five distinct databases, as listed in Table 4 and further detailed for Basal I and Basal II in Additional file 4: Tables S7, S8 and S9. Among the gene-targets, *C10orf2*, *HSD11B1*, *EGR2*, *FBXL5*, *CLEC7A*, *DNMT3B*, *FMO1*, *CTSK* and *PYHIN1* were present in at least two databases. A comparison between miRNA and gene expression levels across subgroups showed significant correlations of hsa-miR-142-5p and *RASSF5*, hsa-miR-142-5p and *TIMP3*, hsa-miR-150 and *MIAT*, and hsa-miR-22 and *TIMP3* in both Basal I and Basal I.

**Table 3:** MicroRNAs differentiating between basal-like breast cancer subgroups

miRNA	Probe ID	*p*-value
hsa-put-miR-92597	CRINCR2000005427	2.8·10^−4^
hsa-miR-361-3p	A_25_P00012305	2.8·10^−4^
hsa-miR-342-3p	A_25_P00012357	4·10^−4^
hsa-miR-140-3p	A_25_P00012177	1.3·10^−4^
hsa-miR-34a	A_25_P00012086	4.9·10^−3^
hsa-miR-22	A_25_P00010204	6.3·10^−3^
hsa-miR-142-5p	A_25_P00014844	2·10^−4^
hsa-miR-142-3p	A_25_P00011016	2.2·10^−3^
hsa-miR-155	A_25_P00012271	6.3·10^−6^
hsa-miR-342-5p	A_25_P00012354	2·10^−7^
hsa-miR-150	A_25_P00014847	8.7·10^−6^
hsa-put-miR-4391	CRINCR2000005084	1.2·10^−4^
hsa-miR-29c	A_25_P00012274	6.7·10^−3^
hsa-miR-29c*	A_25_P00013484	5.6·10^−4^
hsa-miR-29a	A_25_P00012013	4.8·10^−3^
hsa-miR-19b-1*	A_25_P00013163	5.3·10^−4^
hsa-miR-17*	A_25_P00013151	5·10^−4^
hsa-miR-17	A_25_P00013841	1.9·10^−3^
hsa-miR-200c*	A_25_P00013469	1.8·10^−4^

The miRNAs differentially expressed in Basal I and II subgroups are listed in this table, with the corresponding *p*-value in the METABRIC training set. Probes above the mid-line indicate the miRNAs over-expressed in Basal I, while those below are over-expressed in Basal II. Probe IDs correspond to the Agilent platform

**Table 4:** MicroRNAs and corresponding target genes

miRNA	Target
hsa-miR-361-3p	*C3AR1*, *CEBPA*, *GM2A*, *MIAT*, *SURF6*, *TIMP3*
hsa-miR-342-3p	*MXD3*, *PSMG3*, *PTCRA*, *PTPRC*, *TIMP3*
hsa-miR-140-3p	*C10orf2*, *CXCL11*, *KCTD15*, *PNPLA4*, *PRKCSH*, *RRP12*, *STK25*
hsa-miR-34a	*CXCL11*, *DSN1*, *FCGR2A*, *GPR65*, *IKZF3*, *PNPLA4*
hsa-miR-22	*DOK2*, *GM2A*, *HSD11B1*, *MXD3*, *PNPLA4*, *STK25*, *TIMP3*
hsa-miR-142-5p	*C10orf2*, *CD24*, *CEBPA*, *EGR2*, *FBXL5*, *FPR3*, *HSD11B1*, *RASSF5*, *TIMP3*
hsa-miR-142-3p	*CD24*, *EGR2*, *PNPLA4*, *SH3BGRL*
hsa-miR-155	*PSRC1*, *RBFA*
hsa-miR-342-5p	*ASPSCR1*, *CASP4*, *IKZF1*, *PSRC1*
hsa-miR-150	*CCR1*, *EGR2*, *FBXL5*, *MIAT*
hsa-miR-29c	*CLEC7A*, *DNMT3B*, *FCGR2A*, *FMO1*, *KCTD15*, *MIAT*, *TPX2*
hsa-miR-29c*	*GARNL3*, *HJURP*, *MIS18A*
hsa-miR-29a	*CLEC7A*, *DNMT3B*, *FCGR2A*, *FMO1*, *KCTD15*, *MIAT*, *TPX2*
hsa-miR-19b-1*	*CXCR6*, *FCGR2A*, *HSD11B1*, *MXD3*
hsa-miR-17	*AIM2*, *BEND3*, *CEL*, *CTSK*, *EGR2*, *FBXL5*, *PNPLA4*, *PYHIN1*, *SNTB1*, *TIMP3*
hsa-miR-200c*	*DOK2*, *HJURP*, *IL2RA*, *PSRC1*, *RRP12*

The differentially expressed miRNAs and corresponding target genes within the 80-probe signature are listed in this table. The matching targets appeared in at least one of the five databases: *miRBase*, *TarBase*, *PicTar*, *MirTarget2* and *miRanda*. Target genes that were present in at least two databases are underlined

### Copy number aberration profiles further differentiating basal-like subgroups

**Fig. 5:**
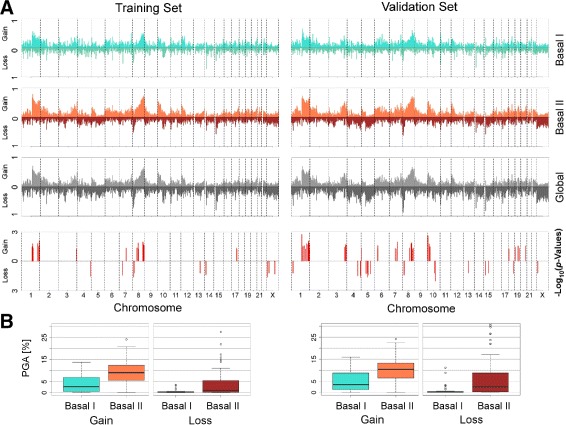
Copy number aberration defined for basal-like subgroups in the METABRIC data set. **a** The CNA information is plotted for 23 chromosomes (including the X chromosome); the percentage of the population showing amplification/gain (Amp) or deletion/loss (Del) were calculated for each cytoband. **b** The boxplots represent the PGA computed for each METABRIC data set

The integrated analysis of CNA has revealed an increasing number of genomic changes from Basal I to Basal II subgroup (Fig. 5) and uncovered cytobands with significant aberrations (binomial test *p*-values below 0.15) in both METABRIC training and validation sets (Table 5). Accordingly, critical gains/amplifications were detected on chromosomes 1q, 3q, 8q, 10p and 17q, and losses/deletions on 4q, 5q, 8p, Xp and Xq. Several of these aberrations have been previously associated with primary breast tumours and cell lines in BLBCs and/or TNBCs studies [20, 47–50].

**Table 5:** Cytobands associated with significant CNA acquisitions

Type	Cytobands	Training set	Validation set
		*p*-value	*p*-value
Gain	1q21.1	0.055	0.0012
	1q22	0.012	0.033
	1q23.1	0.038	0.024
	1q24.1	0.123	0.064
	1q32.3	0.099	0.14
	1q42.11	0.012	0.017
	1q42.12	0.08	0.015
	1q42.13	0.023	0.0059
	1q42.3	0.12	0.03
	1q43	0.0063	0.012
	1q44	0.021	0.049
	3q28	0.044	0.016
	8q13.2	0.044	0.054
	8q21.13	0.14	0.037
	8q22.1	0.092	0.06
	8q22.2	0.097	0.065
	8q23.2	0.12	0.0096
	8q24.11	0.075	0.049
	8q24.21	0.05	0.039
	8q24.22	0.012	0.086
	10p15.3	0.1	0.004
	10p12.32	0.12	0.013
	17q25.1	0.06	0.1
Loss	4q35.1	0.021	0.015
	5q12.2	0.15	0.04
	5q14.3	0.06	0.1
	8p21.2	0.046	0.027
	8p21.1	0.085	0.043
	Xp22.13	0.066	0.046
	Xp21.2	0.049	0.059
	Xq13.3	0.066	0.053
	Xq21.2	0.14	0.12
	Xq21.32	0.066	0.053

Chromosome aberrations in cytobands are classified into two major types: amplifications/gains and deletions/losses. The *p*-values were calculated using the binomial test with respect to the global distribution

Notably, the percent of the genome being altered in the training set for Basal I was 2.74*%* for gains and 0.23*%* for losses; in Basal II it was 9.06 and 1.03*%*, respectively. The Wilcoxon test showed significant heterogeneity among the subgroups for the gains (*p*-value = 1.91·10^−6^) and for losses (*p*-value = 9.55·10^−4^). The same pattern was observed in the validation set for Basal I (3.58*%* for gains and 0.13*%*) and Basal II (10.46*%* for gains and 2.54*%*), also highly significant (Wilcoxon test: *p*-value = 1.11·10^−6^ for gains and *p*-value = 5.37·10^−6^ for losses). The increasing genome instability represented by increasing PGA, plotted in Fig. 5, occurred consistently, from Basal I to Basal II, with the decreasing rates of patients’ survival.

## Discussion

### Survival-related probes defining the molecular signature of basal-like breast cancer subgroups

The basal-like subgroups defined in this study show distinct patterns in terms of tumour molecular profiles, clinicopathological features and patients survival outcomes. The characterisation of BLBCs, considering the two major entities Basal I and Basal II, is supported by the identification of the 80-probe signature, validated across Illumina and Affymetrix platforms in the METABRIC and ROCK cohorts. The importance of this signature, genes and gene-families, is defined by their functionality for each set: G1, G2 and G3. The annotated probes revealed their association with cell cycle and cell division components, immune/inflammatory regulation and metal binding, respectively, and defined Basal I (Immune Active) and Basal II (High Proliferative) subgroups. In Basal I, the over-expression of G2 probes suggests an immune activation and lymphocytic infiltration, particularly regulating tumour growth and patients’ survival. This role has been previously associated with a better prognosis and therapy response [51], and has the potential to stratify basal-like breast cancers. On the other hand, the over-expression of G1 cell cycle-related genes and under-expression of G3 metal binding genes in Basal II impact on cell proliferation rates and energy metabolism. In this case, the cells reproduce at a rate far beyond the common bounds of a controlled cell cycle, concomitantly with other molecular changes in metabolic processes.

The G1 genes *PSMG3*, *HJURP*, *BEND3*, *TPX2*, *RRP12* and *DNMT3B* exhibited the highest centrality values and were over-expressed in the Basal II subgroup. *HJURP*, for instance, plays a central role in the maintenance of newly replicated centromeres and mitotic regulation. Increased levels of this gene in primary tumours and breast cancer cell lines have been previously correlated to decreased disease-free and overall survival [52]. Also involved in the mitotic spindle assembly, *TPX2*, when over-expressed, has been associated with proliferation networks and metastasis enhancement, holding a prognostic value for breast cancer patients [53]. Additionally, the hyperactivity of the DNA methyltransferase enzymes, or the over-expression of *DNMT3B*, has been further reported in BLBCs and TNBCs, where the hypermethylation events were more frequent than in other breast cancer subtypes [54]. Hypermethylated tumours also presented decreased levels of regulatory miRNAs, including hsa-miR-29a and -29b. In particular, the under-expression of hsa-miR-29c has been marked as characteristic of BLBCs, segregating them into two subsets [55], which has been supported by our findings. More studies, however, are required to investigate the biological role of other representative genes, such as *PSMG3*, *BEND3* and *RRP12* in G1.

A number of G2 genes are key regulators of the basal-like tumorigenesis, such as *CXCR6*, *HCST*, *C3AR1*, *GBP4*, *LY96*, *ANKRD22*, *FPR3* and *FCGR2A*. These genes show the highest betweenness centrality and node degree among tumours, and appeared over-expressed in Basal I. In other reports, the *CXCR6* over-expression has been linked to TNBCs, with distinct roles in autoimmunity and cancer [56]. The co-expression of *CXCR6* and *CXCL16*, a chemokine ligand and receptor, has been associated with inflammatory response and cell migration [57, 58]. In addition, high levels of *HCST* [59, 60], *C3AR1* [61], *GBP4* [62], *LY96* [63], *ANKRD22* [64], *FPR3* [65] and *FCGR2A* [66], have also been related to immune activation and/or inflammatory response in tumours; however, their role in basal-like breast malignancies are yet to be uncovered. In our study, the increased expression levels of these probes, among others genes in the signature, has brought new insights on the basal-like tumour origin and progression, and Basal I and Basal II differentiation.

Standard clinical variables such as tumour size, histology and p53 status have also corroborated with the existence of the two basal-like subgroups. Basal I showed the highest frequency of medullary type, whereas Basal II exhibits the largest average of tumour size and highest frequency of p53 mutation. The interpretation of these features, in practice, support the better outcome of patients within Basal I subgroup, when compared to Basal II. Patients’ age, post-menopausal status, tumour grade, NPI and lymph node invasion, on the other hand, are of a limited value for distinguishing the subgroups. Most of these variables reflect the overall tumour aggressiveness and the subtype poor prognosis.

### MicroRNA expression levels differentiating Basal I from Basal II subgroup

This work is the first instance of miRNA data coverage yielding the analysis of basal-like subgroups, which includes patients with matched genomic, transcriptomic and long-term survival data [67]. The miRNAs have showed an important value for differentiating Basal I (15) and Basal II (4). In Basal I, hsa-miR-361-3p, -342-3p, -140-3p, -34a, -22, -142-5p, -142-3p, -155, -342-5p, -150, -29c and -29a presented increased expression relative to Basal II. Overall, hsa-miR-361-3p has been found over-expressed in TNBCs with respect to other subtypes and healthy controls [68]; and used to discriminate *BRCA1/2* mutation carriers and non-carriers tumours [69]. Greater levels of this miRNA, however, have been associated with a protective value in tumour progression [70] and further linked to inflammatory response [71]. In line with our findings, these results contain additional information for the better understanding of basal-like subgroups. Additionally, high levels of hsa-miR-342-5p [72, 73] and -34a [74, 75] have been correlated to breast cancer decreased recurrence and increased survival; whereas low levels have been associated with cell death inhibition and therapy resistance. The hsa-miR-22 [76, 77] and members of the hsa-miR-29 family (-29a, -29b and -29c) [55, 78] – previously identified as tumour suppressors – have also been implicated in increased survival [78] and pointed out as promising prognostic biomarkers [77, 79].

In Basal II, hsa-miR-19b-1, -17 and -200c presented higher expression levels relative to Basal I and control samples. Tumour cells with enhanced expression of hsa-miR-19 (-19a and -19b-1) have been shown to trigger epithelial-mesenchymal transition [80]. Notably, members of the hsa-miR-200 family have been described as major regulators of this biological process. High levels of hsa-miR-200c and -200b have been observed in circulating tumour cells from patients with metastatic breast cancers [81], indicating the prognostic significance of this biological marker [82, 83]. Consistent with these observations, our results demonstrated the recurrent over-expression of hsa-miR-19b-1 and -200c in Basal II, with the worst disease outcome among the two basal-like subgroups. Ultimately, high levels of hsa-miR-17 has been commonly detected in TNBCs [84], associated with cell migration in vitro and metastasis in vivo [85].

The above described miRNAs matched 50 gene-targets from the 80-probe signature. In our study, hsa-miR-200c* and -29c have been associated with *HJURP* expression levels in G1, hsa-miR-19b-1* with *CXCR6* in G2, and hsa-miR-17 with *CTSK* in G3, which are among the most important genes in the signature. None of these associations, however, have been reported in the literature. On the other hand, studies have demonstrated hits on the gene regulation between hsa-miR-142-5p and *CD24* [86], hsa-miR-29 and *DNMT3B* [87, 88], hsa-miR-142-3p and *EGR2* [89], hsa-miR-150 and *EGR2* [90], hsa-miR-34a and *IKZF3* [91], hsa-miR-150 and *MIAT* [92], hsa-miR-342-3p and *PSMG3*[93, 94], hsa-miR-17 and *TIMP3* [95]. Our results further suggested an important correlation between miRNAS and gene expression values in both Basal I and Basal II, identified by this in silico approach. These and other correlations are, however, highly complex and not fully understood. Additional analysis using in vitro and in vivo models are required to validate our achievements.

### Genomic aberrations further characterise Basal II and Basal I subgroups

Basal-like and triple-negative tumours exhibit the highest frequencies of genomic gains and losses in comparison to other breast cancer subtypes [50]. Significant aberrations observed in this study confirmed the genomic instability among basal-like and further differentiated the two subgroups. The most common aberrations delineating Basal II, with respect to Basal I, occurred on the chromosomes 1, 3, 4, 5, 8, 10, 17 and X.

Gains in 1q, 3q, 8q, 10p and 17q have been identified in our analysis and previously reported in triple-negative tumours [48–50]. Overall, gains on chromosome 1q are the most frequent CNAs detected in breast carcinomas and are normally complex and discontinuous [96, 97]. Amplicons of 1q, 8p and 10p have been also described. These amplicons have contributed to the molecular understanding of this disease and, specially, of basal-like intrinsic subtype [98]. For instance, amplifications in 8q21 have been associated with high tumour grade, high levels of Ki67 and other proliferation markers, including *MYC*, *MDM2* and *CCND1* [99]. Gains in 10p have further differentiated triple-negative cancers [48], and in 17q25 have distinguished *BRCA1*-mutated tumours [100].

Losses in 4q, 5q, 8p, Xp and Xq have been defined as key aberrations within basal-like tumours in our analysis and among other breast cancer studies [20, 49]. Frequent losses in 4q and 5q in *BRCA1*-mutated tumours have distinguished them from sporadic neoplasms. In particular, the loss in 5q has impacted the expression of several *BRCA1*-dependent genes involved in DNA repair, such as *RAD17* and *RAD51* [101]. High incidence rates of gains in 5q14 have also been associated with a poor prognosis in BLBCs [102]. Other evidence suggests that aberrations on the X chromosome are common to both *BRCA1*-mutated and sporadic tumours [103].

Overall, these aberrations yielded an additional characterisation of Basal I and Basal II. The increasing PGA, or genome instability, from one subgroup to the other complemented the 80-probe signature via the transcriptomic assessment, which is still considered more representative of cellular processes at the proteomic scale [104]. Although the identified CNA did not show a direct correlation with the 80 probes’ expression levels, generally it may lead to widespread disruptions beyond the proposed signature. Ultimately, the above described gains and losses in cytobands – supported by a range of distinct approaches in the literature – further corroborate the differentiation of basal-like subgroups with divergent clinical features and survival outcomes.

### Consensus on the analysis of basal-like breast cancer subtypes: a literature overview

In this section, we further established a consensus on the description of basal-like subgroups (Basal I and Basal II) by comparing our results with other achievements across the literature [10, 19–21, 31], as per the focus of each study. Notably, most of them have centred on the classification of triple-negative entities, a more heterogeneous group than basal-like. For instance, among the six intrinsic TNBC subtypes defined by Lehmann et al. (2011) [19], three were considered relevant for further comparisons against the proposed basal-like subgroups: the basal-like (BL1 and BL2) and the immunomodulatory (IM). The groups were described based on cell cycle regulation, DNA damage response and immunomodulatory related-genes, respectively. These genes hint to the involvement of similar mechanisms differentiating between Basal I and Basal II, indicating that both classifications are somehow related. Genes (G1) with high node centrality values in Basal II, such as *HJURP* and *TPX2* have been linked to aberrant proliferation networks, cell invasion and metastasis in breast cancer, in line with the definition of BL1 [19]. In addition, genes (G2) defining the Basal I subgroup, including *CXCR6*, *HCST*, *C3AR1*, *GBP4*, *LY96*, *ANKRD22*, *FPR3* and *FCGR2A*, have association with immune activation and inflammatory response, closer to IM [19]. Major regulations involving these genes support the existence of the two subgroups, even though the pool of samples were considerably distinct, BLBCs and TNBCs.

In the recent classification of TNBCs performed by Burstein et al. (2014) [20], two groups were described: the basal-like immune-activated (BLIA) and immune-suppressed (BLIS) subtypes, corresponding to the best and worst prognosis, respectively. In BLIA, tumours display an over-expression of Stat signal transduction molecules and cytokines; in BLIS, high levels of the immunosuppressing molecule *VTCN1*. The mechanisms defining BLIA follow the characteristics of Basal I, and BLIS follows Basal II. For example, Basal I and BLIA [20] contain common genes and/or genes belonging to the same family, such as *CXCL9/10/11/13*, *GBP4/5* and *CD2/24*. Similarly, Jézéquel et al. (2015) [21] identified two relevant subtypes: basal-like with low immune response and high M2-like macrophages (C2), and basal-enriched with high immune response and low M2-like macrophages (C3). The defined basal-like and basal-enriched groups shared evident similarities with Basal II and Basal I, respectively, and corroborated with our study in terms of probe signature and functionality. With regards to the TNBC classification, however, Lehmann et al. (2011) [19], Burstein et al. (2014) [20] and Jézéquel et al. (2015) [21] partially support each other.

An alternative approach to differentiating two subgroups of basal-like – associated with either a low or high risk of disease relapse – has been tested by Hallett et al. (2012) [10], using a 14-gene signature. Among the genes in the signature, *RPL3* and *GPR27* were listed as key markers of relapse, while *RPL36AL* and *GPR65* appeared as variants in the 80 survival-related probes. In the same direction, Sabatier et al. (2011) [31] identified a 28-kinase metagene signature – associated with disease-free survival and immune response – used to divide the BLBCs into two groups: ‘Immune High’ and ‘Immune Low’. This approach revealed key genes, including *IL2RG/B*, *GBP2*, *CCR5/7*, *CXCR3/5/6* and *CXCL9/13*, related to their family members in our signature, such as *IL2RA*, *GBP4*, *CCR1*, *CXCR6* and *CXCL11*. These genes appeared over-expressed in ‘Immune High’ [31] and in Basal I subgroup, when compared to ‘Immune Low’ [31] and Basal II.

Integrating these observations, there is a clear consensus on the segregation of basal-like breast cancers into at least two subgroups. Basal I (Immune Active) show molecular overlaps and phenotypic similarities with BLIA [20], IM [19] and C3 [21]; Basal II (High Proliferative) matched with BLIS [20] and C2 [21]. The comprehensive genomic and transcriptomic characterisation of the two subgroups, provided in this study, will lead to the better understanding of the mechanisms involved in basal-like tumours and to the identification of groups of patients with distinct disease outcome, supported by additional survival features [10, 31]. The latter is crucial for improving the clinical decision-making and for helping tailor treatments that are focused on the immune system manipulation and the cell cycle pathway intervention. In general, tumours with activated immune response have shown a favourable prognosis [15] and are likely to respond to chemotherapy [31], whereas the high proliferative ones have revealed increased risk of metastasis and recurrence [18]. In this context, patients at a low risk should follow more conservative therapies and those at high risk should receive more effective drugs for improving individual response, towards a more personalised medicine.

## Conclusion

Studies have demonstrated that the heterogeneity of BLBCs extends beyond the classic immunohistochemistry. Although several clinicopathological features have been used to discriminate between low- and high-risk patients, the identification of novel biomarkers with prognostic value remains an urgent need for improving breast cancer management. The 80-probe signature defined in this study, associated with varying survival outcomes, contains putative markers of disease progression and represents a promising asset for clinical applications. The integrated assessment of miRNA expression and CNA information, ultimately, contributes towards the definition of more comprehensive profiles of basal-like tumours. The importance of defining groups-at-risk of BLBCs is reflected in the impact of survival-related features in clinical settings and, more importantly, in therapy response.

## Additional files

Additional file 1 Figure S1. Heat map of 400 probes in METABRIC training set. This heat map shows the hierarchical clustering of 115 basal-like samples based on the probe expression values. There are two major clusters: Basal I (turquoise) and Basal II (coral). The 80 probes that best discriminate between the two groups are denoted in orange. The red and blue colours represent relative over- and under-expression, respectively. The expression values are normalised across samples. (JPG 9635.84 kb)

Additional file 2 Basal-like samples classification into Basal I and Basal II, and the centroids defining them. **Tables S1** and **S2** list sample IDs for each basal-like subgroup, Basal I and Basal II; centroids are also provided in **Table S3**. (XLSX 27 kb)

Additional file 3 Functional annotation of G1, G2 and G3 probe sets. These tables contain all probes defined for G1 (**Table S4**), G2 (**Table S5**) and G3 (**Table S6**). The annotation is based on the Database for Annotation, Visualization and Integrated Discovery (DAVID). (XLSX 37 kb)

Additional file 4 Tables S7, S8 and S9. MicroRNAs differentiating between Basal I and Basal II and the corresponding gene targets. Table S7 shows the miRNAs differentially expressed in Basal I and II subgroups, with the corresponding *p*-value in the METABRIC training and validation sets. Tables S8 and S9 list miRNAs and all gene targets for Basal I and Basal II, respectively. (XLSX 69 kb)

## Abbreviations

BLBC: Basal-like breast cancer
BL1: Basal-like 1
BL2: Basal-like 2
BLIS: Basal-like immune-suppressed
BLIA: Basal-like immune-activated
CNA: Copy number aberration
DAVID: Database for annotation, visualization and integrated discovery
EGA: European genome-phenome archive
ER: Oestrogen receptor
HER2: Human epidermal growth factor receptor-2
HREC: Human research ethics committee
IDC: Invasive ductal carcinoma
IDC-med: Invasive ductal carcinoma/medullary carcinoma
ILC: Invasive lobular carcinoma
IM: Immunomodulatory
METABRIC: Molecular taxonomy of breast cancer international consortium
miRNA: microRNA
MS: Menopausal status
MST: Minimum spanning tree
NPI: Nottingham prognostic index
PR: Progesterone receptor
ROCK: Research online cancer knowledgebase
TNBC: Triple-negative breast cancers
