# Supplementary figures and images for: Basal-like breast cancer: molecular profiles, clinical features and survival outcomes
Source: BMC Med Genomics. 2017 Mar 28;10:19. doi: 10.1186/s12920-017-0250-9 (PMC5370447; doi:10.1186/s12920-017-0250-9)

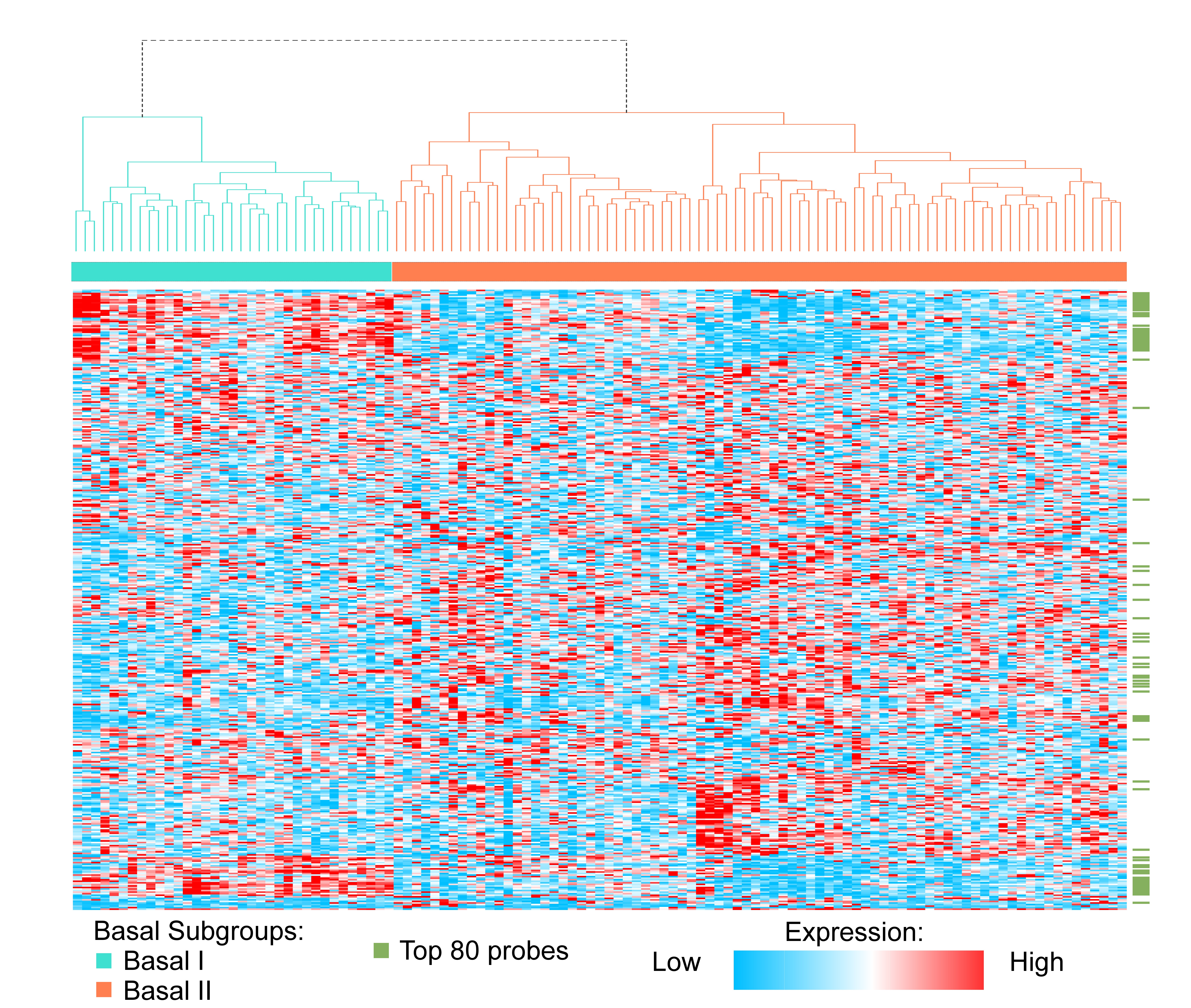

Supplement: Supplementary file 1 — Figure S1. Heat map of 400 probes in METABRIC training set. This heat map shows the hierarchical clustering of 115 basal-like samples based on the probe expression values. There are two major clusters: Basal I (turquoise) and Basal II (coral). The 80 probes that best discriminate between the two groups are denoted in orange. The red and blue colours represent relative over- and under-expression, respectively. The expression values are normalised across samples. (JPG 9635.84 kb) [file 12920_2017_250_MOESM1_ESM.jpg]
